# Oxidant and Antioxidant Parameters’ Assessment Together with Homocysteine and Muscle Enzymes in Racehorses: Evaluation of Positive Effects of Exercise

**DOI:** 10.3390/antiox11061176

**Published:** 2022-06-15

**Authors:** Francesca Arfuso, Maria Rizzo, Claudia Giannetto, Elisabetta Giudice, Roberta Cirincione, Giovanni Cassata, Luca Cicero, Giuseppe Piccione

**Affiliations:** 1 Department of Veterinary Sciences, University of Messina, 98168 Messina, Italy; farfuso@unime.it (F.A.); rizzom@unime.it (M.R.); egiudice@unime.it (E.G.); gpiccione@unime.it (G.P.); 2 Istituto Zooprofilattico Sperimentale della Sicilia “A. Mirri”, 90129 Palermo, Italy; robertacirincione@gmail.com (R.C.); giovanni.cassata@izssicilia.it (G.C.)

**Keywords:** aspartate aminotransferase, athlete horse, creatine kinase, endogenous antioxidants, exercise, lactate dehydrogenase, oxidative stress

## Abstract

This study aimed to evaluate the changes in serum oxidant and antioxidant parameters together with the serum values of homocysteine (Hcy) and muscle enzymes including creatine kinase (CK), aspartate aminotransferase (AST) and lactate dehydrogenase (LDH) in regularly trained athletic horses during official races of 1200, 1600 and 2000 m. Thirty Thoroughbred horses were divided into three groups of 10 subjects each according to the race distance: Group 1, 1200 m race; Group 2, 1600 m race; Group 3, 2000 m race. Blood samples were collected from horses 1 week prior to the race (1WB), on the day of the race at rest (TREST), immediately after the race (TPOST), and after 30 (TPOST30) and 120 (TPOST120) minutes. Serum total proteins, reactive oxygen metabolites (dROMs), thiol antioxidant barrier (SHp), antioxidant barrier (Oxy-ads), Hcy, CK, AST and LDH values were assessed. A two-way repeated measures ANOVA did not show differences referable to the race distance (Group effect) on all investigated parameters (*p* > 0.05). An exercise effect on oxidative stress markers, Hcy and muscle enzymes herein investigated was found in all groups (*p* < 0.001). A Pearson’s test showed dROMs positively correlated with SHp, Oxy-ads and Hcy after exercise (*p* < 0.05). This study suggests that, though well-trained racing horses are subjected to oxidative stress during a race, a proper antioxidant capacity may improve their ability to cope with exercise-induced oxidative stress.

## 1. Introduction

The horse, during its evolution as an animal grazer, developed particular features such as speed and resistance, becoming a great athlete and a valuable animal model for comparative studies on the physiology of human exercise. Indeed, humans and horses are also closely related species because they share a natural aptitude for athletic performance [1]. It is well established that physical exercise affects all the physiological systems in the body and key regulatory systems are called upon to work in concert to re-establish equilibrium [1,2,3]. The failure of the organism to restore balance can threaten an animal’s health status and the physical performance of the athlete [4]. Studies focused on oxidative stress during physical exercise suggest that the degree of oxidative stress and muscle enzyme leakage is dependent on the training level and age of the horse as well as the intensity of exercise [5,6,7]. On the other hand, several studies showed the useful properties of reactive oxygen species in signaling intracellular mechanisms, indicating a biologic redox paradox, and the crucial role of the pathways that control reactive oxygen species’ homeostasis [8]. Though most of the consumed oxygen forms water and carbon dioxide, 1 to 2% of the oxygen, not completely reduced, forms reactive oxygen species [5].

Despite the production of reactive oxygen species by all aerobic cells, natural antioxidant defenses prevent cellular damage by scavenging reactive oxygen species, decreasing the conversion of less reactive to more reactive oxygen species and providing an environment favorable for the activity of other antioxidants [8,9]. When antioxidant defense systems are insufficient or when accumulation of reactive oxygen species becomes chronic, oxidative stress occurs. During exercise, reactive oxygen species’ production may be required for the normal force production in skeletal muscle, for the development of a training-induced adaptation in endurance performance and for the induction of endogenous defense systems [10,11,12].

Despite a desirable increase in reactive oxygen species’ production to ensure optimal muscle contraction [10,13], it is expected that antioxidant enzymes can counter the excess of formed reactive oxygen species to avoid lipid and protein oxidation and, consequently, muscle damage. The oxidative stress response could be influenced by homocysteine (Hcy), a sulfur-containing amino acid [14,15]. Hcy is engaged in the methionine metabolism, nucleic acid biosynthesis, gene methylation, neurotransmission and phospholipid biosynthesis [14]. It has been suggested that Hcy potentiates thrombin production in the endothelial cells [15,16]. Thrombin is a potent activator of a unique group of protease-activated receptors (PARs) belonging to the G protein-coupled receptor family, and the activation of PARs is likely to induce the production of reactive oxygen species, to up-regulate NADPH oxidase and to down-regulate thioredoxin [16] in the endothelial cells. Notably physical activity may modify Hcy levels; however, research examining the impact of exercise on Hcy levels is equivocal. This incoherence in results on exercise-related changes in Hcy levels is partially due to a lack of control for variables including the duration, intensity and mode of exercise that impact Hcy [17,18].

In light of the above considerations, the purpose of the current study was to evaluate the oxidative stress degree by assessing changes in reactive oxygen metabolites (dROMs), the thiol antioxidant barrier (SHp) and antioxidant barrier (Oxy-ads) together with the serum values of homocysteine creatine kinase, aspartate aminotransferase and lactate dehydrogenase in regularly trained athletic horses during official races of 1200, 1600 and 2000 m in order to establish a possible relationship between oxidant/antioxidant parameters and muscle enzymes.

## 2. Materials and Methods

All treatments, housing and animal care reported below were carried out in accordance with the standards recommended by the EU Directive 2010/63/EU for animal experiments.

### 2.1. Animals and Experimental Design

A total of 30 regularly trained male Thoroughbred horses (4 ± 1 years old) with a mean body weight of 433 ± 17 kg, were enrolled in this study with the owner’s informed consent. The animals enrolled in the study came from the same training center, which provides a continuous and well-planned assessment of the state of fitness and health of each animal. Specifically, horses were subjected twice a month to clinical examination, evaluation of the main physiological parameters and blood sampling for hematology and biochemistry analyses at rest conditions. Thus, animals were familiar with blood collection procedures, which did not include animal containment. All the horses enrolled in the study were clinically healthy; they had not been treated with drugs or received medication. Fitness training and general animal care were carried out by professional staff not associated with the research team.

All horses underwent standard training before the races, which consisted of fitness training for 6 days per week, with a rest day on Sunday (Table 1).

All animals were exposed to the same environment condition prior to and during the race. Animals were stabled in individual boxes (3.5 × 3.5 m) and were fed standard rations consisting of hay (first-cut meadow hay, sun cured, late cut, 8 kg/horse/d, 6.9% crude protein on average) and a mixture of cereals (oats and barley, 50% each, about 3.5 kg/horse/d) that was provided three times a day at 08:00, 12:00 and 17:00. Composition mean values of mixture were as follows: dry matter, 95%; moisture, 13%; horses digestible protein, 9.11%; crude protein, 13.05%; crude fiber, 20.7%; crude lipid, 3.42; UFC/kg, 0.80. Water was available ad libitum.

Horses were divided into three groups according to the race distance of competition held in Sicily (37°4′31.71″ N; 15°17′11.71″ E; 20.55 m above sea level): Group 1 (n = 10; 3 years old) competed to 1200 m in 1.16 min, Group 2 (n = 10; 3 ± 1 years old) competed to 1600 m in 1.37 min, Group 3 (n = 10; 3 ± 1 years old) competed to 2000 m in 2.09 min.

**Figure 1:**
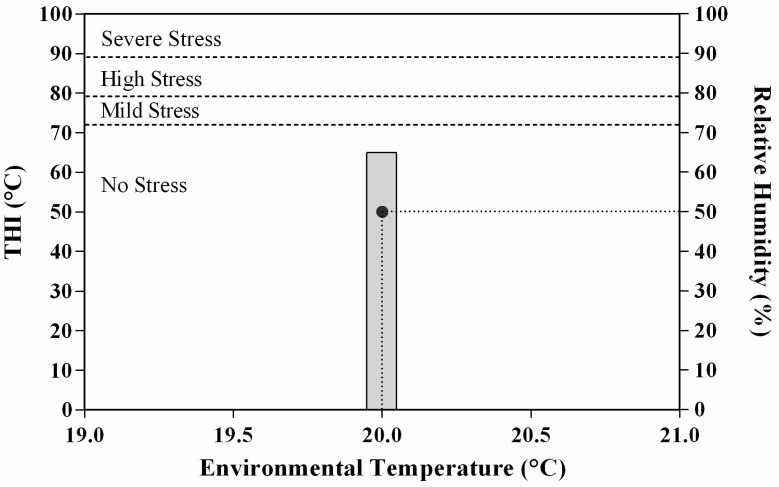
Mean values of ambient temperature, relative humidity and temperature–humidity index (THI) recorded on the day of official races.

Thermal and hygrometric records were carried by means of a data logger (Gemini, UK); they followed the normal seasonal pattern for the location (environmental temperature 20 °C, relative humidity 50%; temperature–humidity index value, THI, of 65 °C) (Figure 1). The THI value was calculated using the U.S. Weather Bureau’s Temperature–Humidity Index Formula [19].

THI (°C) = T ambient + (0.36 − point of steam condensation) + 41.5.

### 2.2. Blood Sampling and Laboratory Analysis

From each horse, blood samples were collected 1 week prior to the race (1WB), the day of the race at rest (TREST), immediately after the race (TPOST), and again after 30 (TPOST30) and 120 (TPOST120) minutes. Blood was collected by jugular venipuncture into two vacutainer tubes (Terumo Corporation, Japan) without anticoagulant agent. The first tube was centrifuged at 3000× *g* for 10 min and, on the obtained sera, the concentration of total proteins, creatine kinase (CK), aspartate aminotransferase (AST), lactate dehydrogenase (LDH), reactive oxygen metabolites (dROMs), antioxidant barrier (Oxy-adsorbent) and thiol antioxidant barrier (SHp) was assessed by means of an automated ultraviolet (UV) spectrophotometer (Slim; SEAC, Florence, Italy). Serum total protein concentration was evaluated by means using the biuret method with commercially available kit (Biosystems S.A., Barcelona, Spain; the protein standard was a bovine albumin, 6.02 g/dL). The serum concentrations of muscle enzymes (CK, AST, LDH) were assessed by commercially available kit (Biosystems S.A., Barcelona, Spain). The values of dROMs, Oxy-Ads and SHp were assessed with the so-called ‘‘spin traps’’ system (Diacron International, Milan, Italy), in which molecules react with free radicals, creating complexes revealed by spectrophotometry [20]. Specifically, the dROMs test assesses the concentration of hydroperoxides (R-OOH), a class of reactive metabolites of the oxygen, in a biological sample (serum, plasma, tissues and cells). The dROMs test is a colorimetric test that assesses the levels of hydroperoxides (R-OOH), the ‘markers’ and ‘amplifiers’ of tissue damage generated by peroxidation of lipids, amino acids, proteins and nucleic acids [21]. In this test, these molecules, after reaction with a properly buffered chromogen, develop a colored derivative, which is photometrically detected. The concentration of ROMs, which directly parallels changes in color intensity, is expressed as Carratelli units (U Carr) where 1 CARR U = 0.08 mg% hydrogen peroxide. Increased values directly correlate to increased levels of oxidative stress. The Oxy-ads test evaluates the ability of plasma to oppose the massive oxidative action of a known title of hypochlorous acid solution [22,23]. In order to assess Oxy-ads, an oxidant solution (1 mL) and a chromogenic mixture (N,Ndiethylparaphenylendiamine) (10 µL) were mixed and the pink-colored complex was read immediately. Decreased values directly correlate with the injury severity of ‘plasma barrier to oxidation’. When the ‘excess’ of radicals of hypochlorous acid after massive oxidation is high, the plasma barrier is reduced and vice versa. The SHp test is a colorimetric determination of plasma/serum thiol antioxidant barrier, which opposes peroxidative processes inhibiting both alkoxyl and hydroxyl radicals [24]. This test is based on the ability of thiol groups to develop a colored complex when reacted with DTNB (5,5-dithiobys-2-nitrobenzoyc acid). In order to assess SHp, a buffer solution (pH 7.6) (1 mL) and a chromogenic mixture (DTNB) (20 µL) were mixed with serum (50 µL). The ‘titer’ of thiols directly parallels color intensity. Decreased values directly correlate with lowered efficacy of thiols antioxidant barrier. All samples were analyzed in duplicate. Samples exhibited parallel displacement to the standard curve. The overall intra- and inter-assay coefficients of variation were calculated as <5%. The second tube was centrifuged at 2000× *g* for 20 min and, on the obtained sera, the total homocysteine (Hcy) values were determined by high performance liquid chromatography (HPLC—Agilent 1100, BIO-RAD) with fluorometric detection and isocratic elution. This methodology involves three steps, namely, a reduction in thiol groups using TCEP (tris(CarboxyEthyl)Phosphine), protein precipitation, and derivatization with SBD-F (7-fluorobenzene-2-oxy-1,3-diazolic-4-ammonium sulfate). The HPLC system used was a Shimadzu apparatus with an SIL-10ADvp automatic sample injector and an RF-10AXL fluorescence detector. Chromatographic separation was performed by using a C18 model Shim-pack CLC-ODS column (4.6 × 150 mm with 5.0 µm microparticles). The fluorescence of the separated compounds was measured with a detector adjusted for excitation at 385 nm and emission at 515 nm. The total Hcy concentrations were calculated with a calibration curve by using known amino acid concentrations and cystamine as the internal standard.

### 2.3. Statistical Analysis

All data were tested for normality of distribution using the Kolmogorov–Smirnov test. All data resulted normally distributed (*p* > 0.05) and parametric statistical analysis was performed.

Two-way repeated measures analysis of variance (ANOVA) was applied to determine statistical significant effect of exercise and time on serum oxidative markers (dROMs, SHp and Oxy-ads), Hcy, CK, LDH, AST and total proteins in enrolled horses. Bonferroni multiple comparison test was applied for post hoc comparison. Pearson’s correlation coefficients were computed to assess the possible relationship between the serum values of dROMs and the other considered oxidative markers (SHp and Oxy-ads), Hcy and muscle enzymes (CK, LDH and AST) in horses throughout the monitoring period. A linear regression model (y = a + bx) was applied to determine the degree of correlation between these variables in studied animals.

*p*-values < 0.05 were considered statistically significant. The statistical analysis was performed using the software Prism v. 9.00 (Graphpad Software Ltd., San Diego, CA, USA, 2020).

## 3. Results

**Figure 2:**
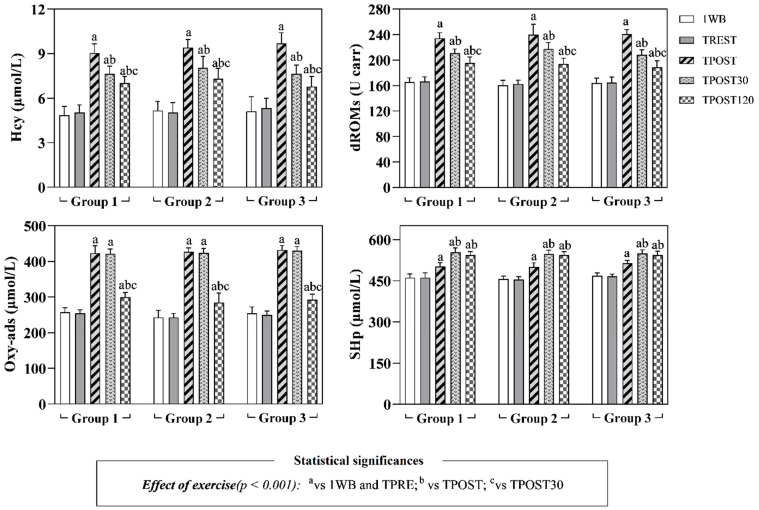
Mean values ± standard deviation (±SD) of serum reactive oxygen metabolites (dROMs), thiol antioxidant barrier (SHp), antioxidant barrier (Oxy-ads) and homocysteine (Hcy) measured 1 week prior to the race (1WB), the day of the race at rest (TREST), immediately after the race (TPOST), and after 30 (TPOST30) and 120 min (TPOST120) in Thoroughbred racehorses belonging to Group 1 (1200 m race), Group 2 (1600 m race) and Group 3 (2000 m race).

**Figure 3:**
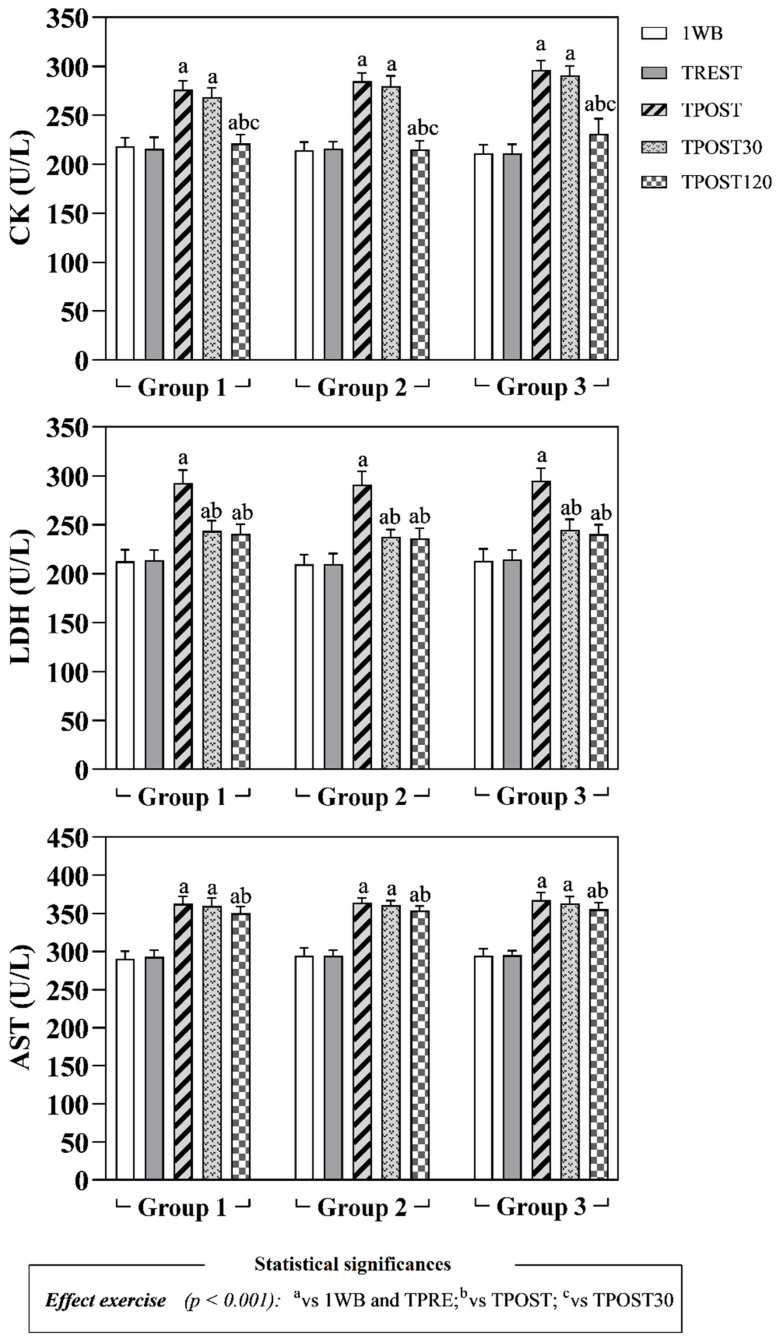
Mean values ± standard deviation (±SD) of serum creatine kinase (CK), aspartate aminotransferase (AST) and lactate dehydrogenase (LDH) measured 1 week prior to the race (1WB), the day of the race at rest (TREST), immediately after the race (TPOST), and after 30 (TPOST30) and 120 min (TPOST120) in Thoroughbred racehorses belonging to Group 1 (1200 m race), Group 2 (1600 m race) and Group 3 (2000 m race).

**Figure 4:**
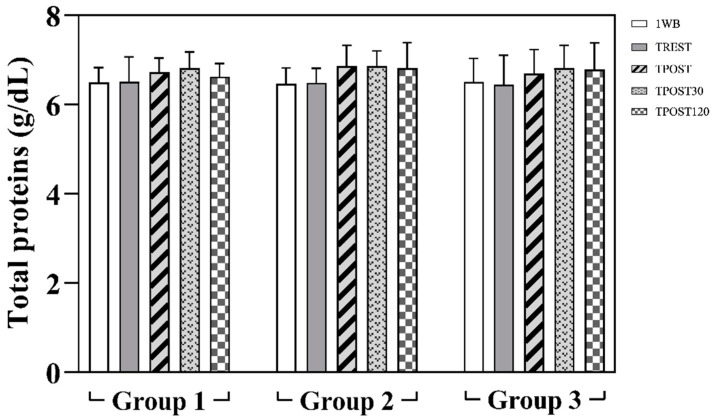
Mean values ± standard deviation (±SD) of serum total proteins measured 1 week prior to the race (1WB), the day of the race at rest (TREST), immediately after the race (TPOST), and after 30 (TPOST30) and 120 min (TPOST120) in Thoroughbred racehorses belonging to Group 1 (1200 m race), Group 2 (1600 m race) and Group 3 (2000 m race).

The obtained data are expressed as mean values ± standard deviation (±SD). Statistical analysis showed no significant differences in the concentration of all parameters measured in horses belonging to all groups 1 week before the beginning of the experimental procedure (1WB) and before the official races, at rest condition (TPRE) (*p* > 0.05). The application of a two-way ANOVA for repeated measures did not show any differences referable to the race distance (Group effect) on all oxidative stress markers, Hcy, muscle enzymes (i.e., CK, LDH, AST) and total proteins (*p* > 0.05, Figure 2, Figure 3 and Figure 4). A significant effect of exercise (*p* < 0.001) on oxidative stress markers (dROMs, SHp and Oxy-ads), Hcy, CK, LDH and AST was found in all investigated Groups, whereas the values of total proteins did not change throughout the monitoring period (*p* > 0.05). Specifically, as shown in Figure 2, higher values of dROMs, SHp, Oxy-ads and Hcy were found after exercise (TPOST, TPOST30 and TPOST120) than 1WB and TREST in Groups 1, 2 and 3 (*p* < 0.001). Moreover, dROMs and Hcy values decreased at TPOST30 and TPOST120 with respect to TPOST, and at TPOST120 compared to TPOST30 in all study groups (*p* < 0.001). Lower Oxy-ads values were found at TPOST120 than at TPOST and TPOST30 in all groups (*p* < 0.001). Contrariwise, higher SHp values were found at TPOST30 and TPOST120 than TPOST in horses belonging to Groups 1, 2 and 3 (*p* < 0.001). Higher CK values were found at TPOST, TPOST30 and TPOST120 than at 1WB and TPRE, whereas this muscle enzyme showed a lower concentration at TPOST120 than at TPOST and TPOST30 in Groups 1, 2 and 3 (*p* < 0.001) (Figure 3).

**Figure 5:**
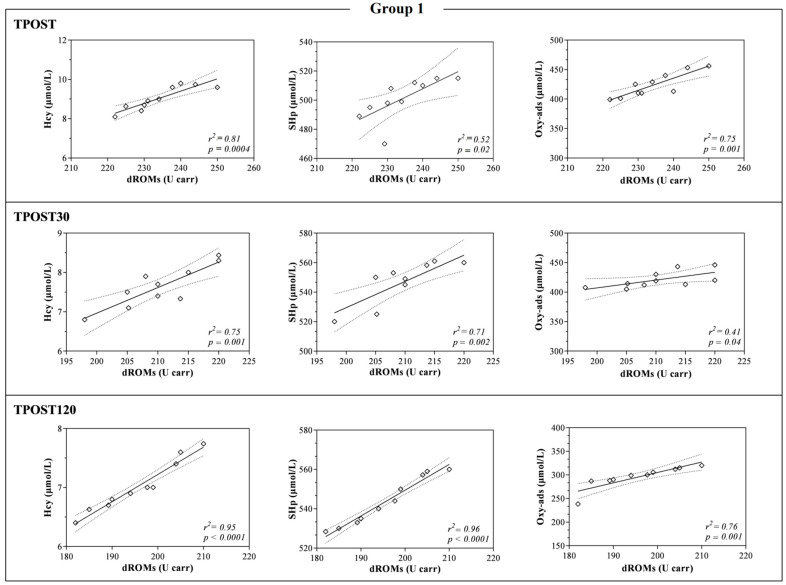
Linear regression values obtained between the serum concentration of reactive oxygen metabolites (dROMs) and the values of homocysteine (Hcy), thiol antioxidant barrier (SHp) and antioxidant barrier (Oxy-ads) obtained immediately after the race (TPOST), and after 30 (TPOST30) and 120 min (TPOST120) in Thoroughbred racehorses competing in 1200 m race (Group 1).

**Figure 6:**
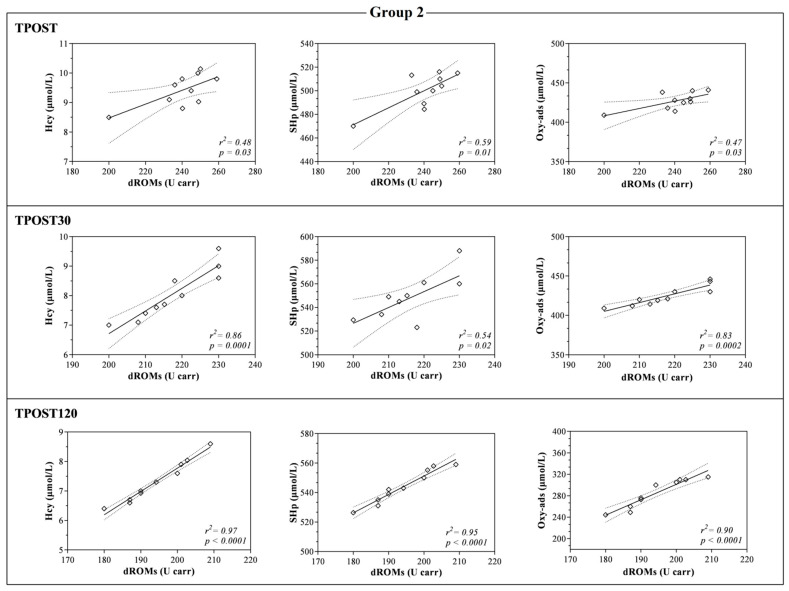
Linear regression values obtained between the serum concentration of reactive oxygen metabolites (dROMs) and the values of homocysteine (Hcy), thiol antioxidant barrier (SHp) and antioxidant barrier (Oxy-ads) obtained immediately after the race (TPOST), and after 30 (TPOST30) and 120 min (TPOST120) in Thoroughbred racehorses competing in 1600-m race (Group 2).

**Figure 7:**
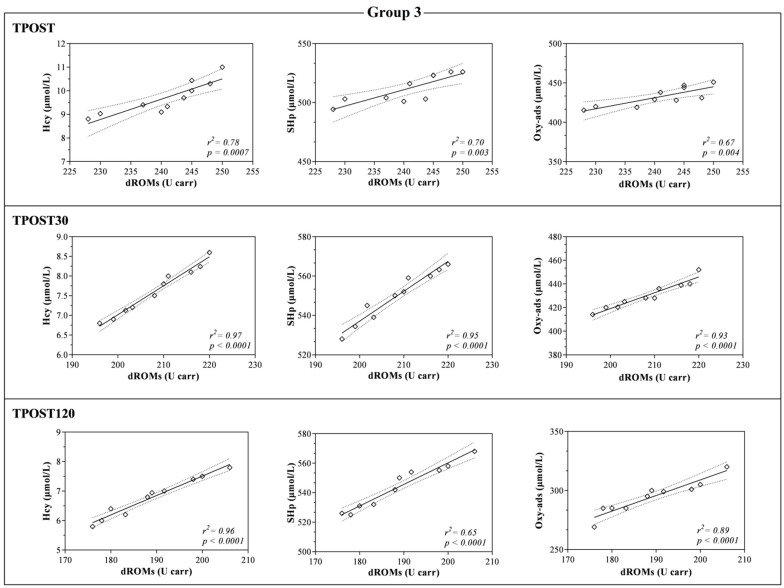
Linear regression values obtained between the serum concentration of reactive oxygen metabolites (dROMs) and the values of homocysteine (Hcy), thiol antioxidant barrier (SHp) and antioxidant barrier (Oxy-ads) obtained immediately after the race (TPOST), and after 30 (TPOST30) and 120 min (TPOST120) in Thoroughbred racehorses competing in 2000-m race (Group 3).

The LDH and AST values were higher at TPOST, TPOST30 and TPOST120 than 1WB in each group (*p* < 0.001, Figure 3). Furthermore, LDH showed a statistically significant decrease at TPOST30 and TPOST120 with respect to the values recorded immediately after exercise (TPOST) in each group (*p* < 0.001), whereas AST statistically decreased in each group at TPOST120 in comparison to TPOST (*p* < 0.05, Figure 3). The Pearson’s test showed a positive correlation between the values of dROMs and the concentrations of SHp, Oxy-ads and Hcy after exercise (TPOST, TPOST30 and TPOST120), whereas no significant correlation between dROMs and the values of CK, AST and LDH was found (Table 2). These findings were confirmed by the linear regression model results (Figure 5, Figure 6 and Figure 7).

## 4. Discussion

It is well established that physical exercise leads to an increase in reactive oxygen species production in sport horses [25,26,27,28], therefore, the inclusion of oxidative stress monitoring in the health management of athletes might be argued. The results obtained in the present study highlight the influence of exercise on oxidant and antioxidant indices during official races in athletic horses without an effect of the different length of the race course (group effect) in all investigated parameters. The serum values of total proteins measured in each horse did not statistically change throughout the monitoring period, falling within the physiological range established for adult horses [29,30]. This result indicates that the animals were adequately nourished to maintain the serum levels of total proteins in all groups.

Increased dROMs values were found after exercise in each studied group, which highlights the occurrence of the oxidative process during exercise [31,32]. The increase in dROMs could be related to the rise of oxygen-carrying erythrocytes as well as of hemoglobin concentration due to an increase in the blood cell–volume ratio known to occur during exercise [31]. Higher dROMs after exercise may also be explained by the increase in glucocorticoids and catecholamine associated with stress, which, as previously demonstrated [26], contributes to the generation of reactive oxygen species. Furthermore, the exercise-related changes in the oxidant/antioxidant equilibrium could be due to increased mitochondrial electron transport within muscle cells. Notably, the highest dROMs values were found immediately after the race followed by a gradual decrease 30 and 120 min after the end of exercise in all investigated groups. The same trend was observed for Hcy concentration. In agreement with these results, previous studies showed higher Hcy concentrations after acute exercise, a training period or after a specific sport competition [33,34]. High Hcy concentration plays a role in skeletal muscle weakness [18], indeed it is associated with physical function decline. Other authors demonstrated reduced Hcy concentrations after a training period [35,36].

As previously suggested [32], oxidative stress induces an increase in the uptake and utilization of amino acids including cysteine and methionine. Methionine is converted to S-adenosylmethionine that is transformed to S-adenosylhomocysteine and, ultimately, it is converted to Hcy [32]. Therefore, according to previous observations [32,33,34], and to the results obtained in the current study, it could be stated that the oxidative stress induced by exercise increases serum Hcy concentration as highlighted by the strong positive correlation herein found between dROMs and Hcy at each time point after exercise. According to the results obtained in the current study, increased values of Oxy-ads and SHp were observed after the race in each studied group, suggesting an antioxidant compensation following exercise. This assumption seems to be strengthened by the positive correlation between the dROMs values and the Oxy-ads and SHp concentrations found in horses belonging to each group after the exercise.

The results gathered in the current study showed a dynamic change in serum CK, AST and LDH values in each group after exercise compared to the concentration measured during the rest condition. In particular, as previously observed [37], all horses showed increased values of CK and AST immediately after and 30 min after the race compared to rest. The CK concentration decreased 120 min after the race as compared to TPOST and TPOST30, while AST values persisted to be high, probably due to the long half-life of AST in the serum [38]. The serum concentration of CK measured in horses herein investigated were lower than the values obtained in previous studies carried out on racehorses, and this difference could be attributable to the difference in exercise intensity. It is well established that during physical exercise there is an increase in CK in horses; however, this increase is not necessarily an index of insufficient fitness or muscular fatigue [23]. The increase in the serum concentrations of muscle enzymes such as CK and AST may be expected due to intense muscle effort [37]. In particular, increased AST levels during physical activity could be due to a loss and/or modification of the muscle fiber membrane with a temporarily increased permeability [39]. The significant increase in serum LDH values after the race compared to the rest condition may be due to a rise in mitochondrial membrane permeability, associated with the maximal physical exercise undertaken [23], instead of muscular lesions. The concentration of muscle enzymes considered in the current study (i.e., CK, AST and LDH) was not correlated with dROMs values throughout the monitoring period (both at rest and after races), suggesting that, though physical exercise led to an increase in dROMs production, the serum levels of reactive oxidation species were not such as to cause damage at the muscular level. It is important to point out that the horses herein investigated were regularly trained with a standard training program. As previously demonstrated, tissues seem to increase their antioxidant defense when exposed to chronic activation and, therefore, the application of adequate training programs could improve performance by increasing the antioxidant defense in tissues exposed to exercise [40]. It would appear that, though the role of increased ROS production in exercise-induced damage is controversial, the increased production of ROS seems to have an important role for the adaptation of skeletal muscle following exercise [41].

## 5. Conclusions

This study confirms that maximal exercise induces an increase in dROMs, Oxy-ads and SHp values in Thoroughbred racing horses at 1200, 1600 and 2000 m, and it suggests that the oxidative stress induced by exercise increases serum Hcy concentration as highlighted by the strong positive correlation herein found between dROMs and Hcy at each time point after exercise. Though regularly trained racehorses are susceptible to oxidative stress during a race, on the other hand, a strong antioxidant capacity may improve their ability to cope with exercise-induced oxidative stress as strengthened by the positive correlation found between the concentration of dROMs and the values of Oxy-ads and SHp in horses belonging to each group after the exercise. Furthermore, the increase in serum CK, LDH and AST values observed after exercise compared to the rest condition in all investigated horses may be expected due to the intense muscle effort. Since the concentrations of these enzymes were not correlated with dROMs values both at rest and after races it can be assumed that the reactive oxidation species generated following exercise are not responsible for damage at the muscular level in the regularly trained horses investigated in the current study. Further studies in athletic horses of different breeds and categories are needed to outline a complete picture of the activity of several proteins, enzymatic or non-enzymatic, operating in different steps of free radicals’ neutralization during exercise, thus helping to enrich the current knowledge on this field to improve both the health status and the physical performance of the athlete.

## Figures and Tables

**Table 1:** Schedules of training program performed by horses belonging to Group 1 (1200 m/race; n = 10), Group 2 (1600 m/race; n = 10) and Group 3 (2000 m/race; n = 10).

*Days of Week*	*Gait*	*Duration (minutes)*		
		Group 1	Group 2	Group 3
I, III and V	Walk	5	5	5
	Trot	15	10	10
	Canter (360 m/min)	20	25	30
	Gallop (600 m/min)	6	5	5
	Walk	5	5	5
II, IV and VI	Walk	5	5	5
	Trot	15	20	20
	Canter (360 m/min)	15	20	20
	Gallop (600 m/min)	3	3	3
	Walk	5	5	5
VII	Rest	-	-	-

**Table 2:** Coefficients of correlation between the serum values of reactive oxygen metabolites (dROMs) and the levels of thiol antioxidant barrier (SHp) and antioxidant barrier (Oxy-ads), homocysteine (Hcy), creatine kinase (CK), lactate dehydrogenase (LDH) and aspartate aminotransferase (AST) obtained from racehorses competing in official races of 1200 (Group 1), 1600 (Group 2) and 2000 m (Group 3). Blood samples were collected from each horse 1 week prior to the race (1WB), the day of the race at rest (TREST), immediately after the race (TPOST), and after 30 (TPOST30) and 120 min (TPOST120). *p*-values < 0.05 were considered statistically significant.

		Parameters					
***GROUP 1***		***Hcy***	***SHp***	***Oxy-ads***	***CK***	***LDH***	***AST***
		(μmol/L)	(μmol/L)	(μmol/L)	(U/L)	(U/L)	(U/L)
*dROMs* (*U Carr*)	1WB	r = −0.09	r = −0.003	r = −0.04	r = 0.27	r = 0.26	r = 0.53
		*p* = 0.80	*p* = 0.99	*p* = 0.93	*p* = 0.46	*p* = 0.47	*p* = 0.11
	TPRE	r = −0.40	r = 0.24	r = −0.22	r = −0.26	r = −0.002	r = 0.34
		*p* = 0.26	*p* = 0.51	*p* = 0.54	*p* = 0.47	*p* = 0.99	*p* = 0.34
	TPOST	r = 0.90	r = 0.72	r = 0.87	r = 0.13	r = 0.03	r = 0.10
		*p* = 0.004	*p* = 0.02	*p* = 0.001	*p* = 0.72	*p* = 0.94	*p* = 0.78
	TPOST30	r = 0.86	r = 0.85	r = 0.64	r = 0.35	r = 0.16	r = 0.15
		*p* = 0.001	*p* = 0.002	*p* = 0.04	*p* = 0.34	*p* = 0.66	*p* = 0.67
	TPOST120	r = 0.98	r = 0.98	r = 0.87	r = 0.22	r = −0.20	r = 0.27
		*p* < 0.0001	*p* < 0.0001	*p* = 0.001	*p* = 0.54	*p* = 0.58	*p* = 0.45
***GROUP 2***		***Hcy***	***SHp***	***Oxy−ads***	***CK***	***LDH***	***AST***
		(μmol/L)	(μmol/L)	(μmol/L)	(U/L)	(U/L)	(U/L)
*dROMs* (*U Carr*)	1WB	r = −0.43	r = 0.04	r = 0.58	r = 0.20	r = 0.33	r = 0.25
		*p* = 0.22	*p* = 0.92	*p* = 0.08	*p* = 0.57	*p* = 0.35	*p* = 0.48
	TPRE	r = −0.33	r = 0.35	r = −0.32	r = −0.53	r = −0.43	r = 0.42
		*p* = 0.35	*p* = 0.33	*p* = 0.36	*p* = 0.09	*p* = 0.21	*p* = 0.23
	TPOST	r = 0.69	r = 0.77	r = 0.68	r = −0.04	r = −0.20	r = −0.16
		*p* = 0.03	*p* = 0.009	*p* = 0.03	*p* = 0.91	*p* = 0.57	*p* = 0.67
	TPOST30	r = 0.93	r = 0.73	r = 0.91	r = −0.07	r = 0.08	r = 0.38
		*p* = 0.0001	*p* = 0.02	*p* = 0.0002	*p* = 0.84	*p* = 0.82	*p* = 0.27
	TPOST120	r = 0.99	r = 0.97	r = 0.95	r = 0.13	r = −0.21	r = −0.22
		*p* < 0.0001	*p* < 0.0001	*p* < 0.0001	*p* = 0.73	*p* = 0.56	*p* = 0.57
***GROUP 3***		***Hcy***	***SHp***	***Oxy−ads***	***CK***	***LDH***	***AST***
		(μmol/L)	(μmol/L)	(μmol/L)	(U/L)	(U/L)	(U/L)
*dROMs* (*U Carr*)	1WB	r = −0.24	r = −0.37	r = −0.08	r = 0.51	r = 0.44	r = 0.37
		*p* = 0.50	*p* = 0.30	*p* = 0.83	*p* = 0.13	*p* = 0.21	*p* = 0.30
	TPRE	r = 0.10	r = −0.12	r = −0.18	r = 0.002	r = −0.35	r = 0.22
		*p* = 0.79	*p* = 0.73	*p* = 0.62	*p* = 0.99	*p* = 0.31	*p* = 0.54
	TPOST	r = 0.88	r = 0.83	r = 0.81	r = 0.03	r = 0.58	r = 0.31
		*p* = 0.0007	*p* = 0.003	*p* = 0.004	*p* = 0.93	*p* = 0.08	*p* = 0.39
	TPOST30	r = 0.99	r = 0.97	r = 0.96	r = 0.60	r = −0.04	r = 0.40
		*p* < 0.0001	*p* < 0.0001	*p* < 0.0001	*p* = 0.07	*p* = 0.90	*p* = 0.25
	TPOST120	r = 0.98	r = 0.97	r = 0.94	r = −0.05	r = −0.14	r = 0.08
		*p* < 0.0001	*p* < 0.0001	*p* < 0.0001	*p* = 0.90	*p* = 0.70	*p* = 0.83

## Data Availability

Data is contained within the article.
